# Genetic Regulation of GA Metabolism during Vernalization, Floral Bud Initiation and Development in Pak Choi (*Brassica rapa* ssp. *chinensis* Makino)

**DOI:** 10.3389/fpls.2017.01533

**Published:** 2017-09-30

**Authors:** Mengya Shang, Xueting Wang, Jing Zhang, Xianhui Qi, Amin Ping, Leiping Hou, Guoming Xing, Gaizhen Li, Meilan Li

**Affiliations:** ^1^College of Horticulture, Shanxi Agricultural University Taigu, China; ^2^Vegetables Research Institute, Shanxi Academy of Agriculture Sciences Taiyuan, China

**Keywords:** pak choi, vernalization, gibberellins metabolism, expression profiles, gene

## Abstract

Pak choi (*Brassica rapa* ssp. *chinensis* Makino) is a representative seed vernalization vegetable and premature bolting in spring can cause significant economic loss. Thus, it is critical to elucidate the mechanism of molecular regulation of vernalization and floral bud initiation to prevent premature bolting. Gibberellin (GA) is the key plant hormone involved in regulating plant development. To gain a better understanding of GA metabolism in pak choi, the content of GA in pak choi was measured at different stages of plant development using enzyme-linked immunosorbent assay. The results showed that the GA content increased significantly after low-temperature treatment (4°C) and then decreased rapidly with vegetative growth. During floral bud initiation, the GA content increased rapidly until it peaked upon floral bud differentiation. To elucidate these changes in GA content, the expression of homologous genes encoding enzymes directly involved in GA metabolism were analyzed. The results showed that the changes in the expression of four genes involved in GA synthesis (Bra035120 encoding ent-kaurene synthase, Bra009868 encoding ent-kaurene oxidase, Bra015394 encoding ent-kaurenoic acid oxidase, and Bra013890 encoding GA20-oxidase) were correlated with the changes in GA content. In addition, by comparing the expression of genes involved in GA metabolism at different growth stages, seven differentially expressed genes (Bra005596, Bra009285, Bra022565, Bra008362, Bra033324, Bra010802, and Bra030500) were identified. The differential expression of these genes were directly correlated with changes in GA content, suggesting that these genes were directly related to vernalization, floral bud initiation and development. These results contribute to the understanding of the molecular mechanism of changes in GA content during different developmental phases in pak choi.

## Introduction

Pak choi (*Brassica rapa* ssp. *chinensis* Makino) is a cruciferous vegetable and is representative plant that require vernalization. After exposure to low temperatures for a period of time, the plant blossoms under high temperatures and long days. A cultivar with low chilling requirements bolts easily; thus, premature bolting occurs frequently in spring and causes significant reduction in yield and quality (Liu et al., 2009). Therefore, it is crucial to study the molecular regulation of vernalization to prevent bolting in pak choi and possibly other cruciferous vegetables.

Plant hormones play an important role in vernalization and flowering (Aryal and Ming, 2014). Among the six known types plant hormones, gibberellin (GA) is particularly significant (Galvão et al., 2012). GA can partially replace long-day or low temperature to promote flowering in plants (Pearce and Dubcovsky, 2013; Hu et al., 2016). However, the phytohormone GA was shown to play duel, opposing roles in *Arabidopsis*. GA promoted the termination of vegetative development, but it inhibited flower formation (Yamaguchi et al., 2014). Treatment with GA has been shown to promote the flowering of pak choi (Hou et al., 2009; Yu et al., 2009). Therefore, studying the regulation of GA on flowering in pak choi is important.

The biosynthetic and deactivation pathways of GA have been studied in great detail (Liu et al., 2005). A variety of enzymes including ent-copalyl diphosphate synthase (CPS), ent-kaurene synthase (KS), ent-kaurene oxidase (KO), ent-kaurenoic acid oxidase (KAO), GA13-oxidase (GA13ox), GA20-oxidase (GA20ox), GA3-oxidase (GA3ox), and GA2-oxidase (GA2ox), participate in these pathways (Yu et al., 2002; Zhang et al., 2011). In *Arabidopsis*, CPS, KS and KO are encoded by single genes (*GA1, GA2*, and *GA3*, respectively), whereas GA20ox and GA3ox are encoded by small gene families named *GA4* and *GA5*, respectively (Jiang et al., 2008).

The genes involved in GA metabolism have been isolated in a few plant species, including grape (Wang et al., 2012), *Arabidopsis thaliana* (Phillips et al., 1995), tomato (Rebers, 1999), *Camellia lipoensis* (Xiao et al., 2016), tomato (Shen et al., 2016), and pumpkin (Yamaguchi et al., 1996). Although these genes were found to affect the synthesis of GA, their expression patterns were different. In tomato, the expression of the *GA1-*homologous gene *LeCPS* increased during the early stage of floral bud development (Rebers, 1999). In pumpkin, *GA2* was detected high transcription in the vegetative tissues (Yamaguchi et al., 1996). *GA3* from *Arabidopsis* was expressed in inflorescence tissues (Helliwell et al., 1998). *Nty*, the *GA4* ortholog in tobacco, was expressed in specific parts of the floral buds and floral organs (e.g., tapetum) (Yamaguchi et al., 1996; Itoh et al., 1999). *GA5* was expressed in the stem and inflorescence of *Arabidopsis* during pod development (Phillips et al., 1995). These findings indicated that GA plays an important role in different stages of plant flowering. However, the process of GA metabolism during vernalization, floral bud initiation and development in pak choi remains poorly understood. In this study, the GA content in pak choi was measured at different growth stages, and the expression of gene encoding enzymes directly involved in GA metabolism was analyzed, which elucidated the molecular regulation mechanism of GA metabolism during vernalization, floral bud initiation and development in pak choi.

## Materials and methods

### Plant materials and growth conditions

Early-bolting pak choi inbred line “75^#^” was used in this study and was provided by the Institute of Vegetable Research, Shanxi Academy of Agricultural Sciences. The experiment included two treatments: low-temperature treatment and control. For the former (designated as V), the germinating seeds were kept at 4°C for 20 days. In the control (designated as CK), the seeds were germinated at 25°C. Seedlings with the same size were transplanted to trays with 50 holes full of substrate at the same time. The plants were then cultivated using traditional methods.

### Sample collection

After low-temperature treatment, the shoot apices were collected and designated as V0 and CK0 for the low-temperature treatment and control groups, respectively. On 10 days (vegetative growth), 15 days (immediately differentiation) and 16 days (floral bud differentiation stage 1) after transplantation, shoot apices were also collected from both groups, the low-temperature treatment and control samples collected at these times were named as V10, CK10, V15, CK15, V16, and CK16, respectively. Each 0.2 g sample was used to determine the GA content with three biological replicates. Each 0.1 g sample was used for RNA extraction. All samples were frozen in liquid nitrogen and stored at −80°C.

### Measurement of GA content

The GA content at different developmental stages of pak choi were measured by enzyme-linked immunosorbent assay using the method described by Deng et al. (2008). Two types of GA (bioactive GA_1_ and GA_3_) were measured.

### Total RNA extraction and cDNA library construction, sequencing, gene expression analysis, and functional annotation

Total RNA was extracted using an RNeasy Plant Mini Kit according to the manufacturer's instructions (QIAGEN, 74903). The samples used for sequencing were V0, CK0, V10, V15, CK16, and V16. All reads for each sequencing sample were deposited into GenBank, and the SRA accession numbers are SRP111574 (for V10), SRP064332 (for V0, CK0, V15, and CK16) (Sun et al., 2015) and SRP075755 (for V16) (Song et al., 2017; http://www.ncbi.nlm.nih.gov/sra). cDNA library construction and sequencing were performed by Biomarker Technologies Co., Ltd, Beijing, China. The methods of sequencing, expression analysis and functional annotation were previously described by Sun et al. (2015).

### Quantitative real-time PCR

To validate the RNA sequencing results, quantitative real-time PCR (qRT-PCR) was performed using gene-specific primers for four randomly selected genes. Primer3 software was used to design specific primers. The *ACTIN* gene of pak choi was used as a reference gene. The RNA was extracted using an RNeasy Plant Mini Kit (QIAGEN, 74903). First-strand cDNA was obtained using a PrimeScript® RT reagent kit (Perfect Real Time; TaKaRa, RR037A). qRT-PCR was carried out using SYBR® Premix Ex TaqTM II (Tli RNaseH Plus; TaKaRa, RR820A) with an ABI 7500 instrument. The 25-μl reaction mixture included 20 ng cDNA. The program was as follows: 94°C for 1 min; and 40 cycles of 94°C for 30 s, 55°C for 30 s, and 72°C for 30 s. The relative expression levels were calculated using the 2^−ΔΔCt^ method (Stanko et al., 2014).

## Results

### GA contents of pak choi shoot apices at different growth stages

**Figure 1:**
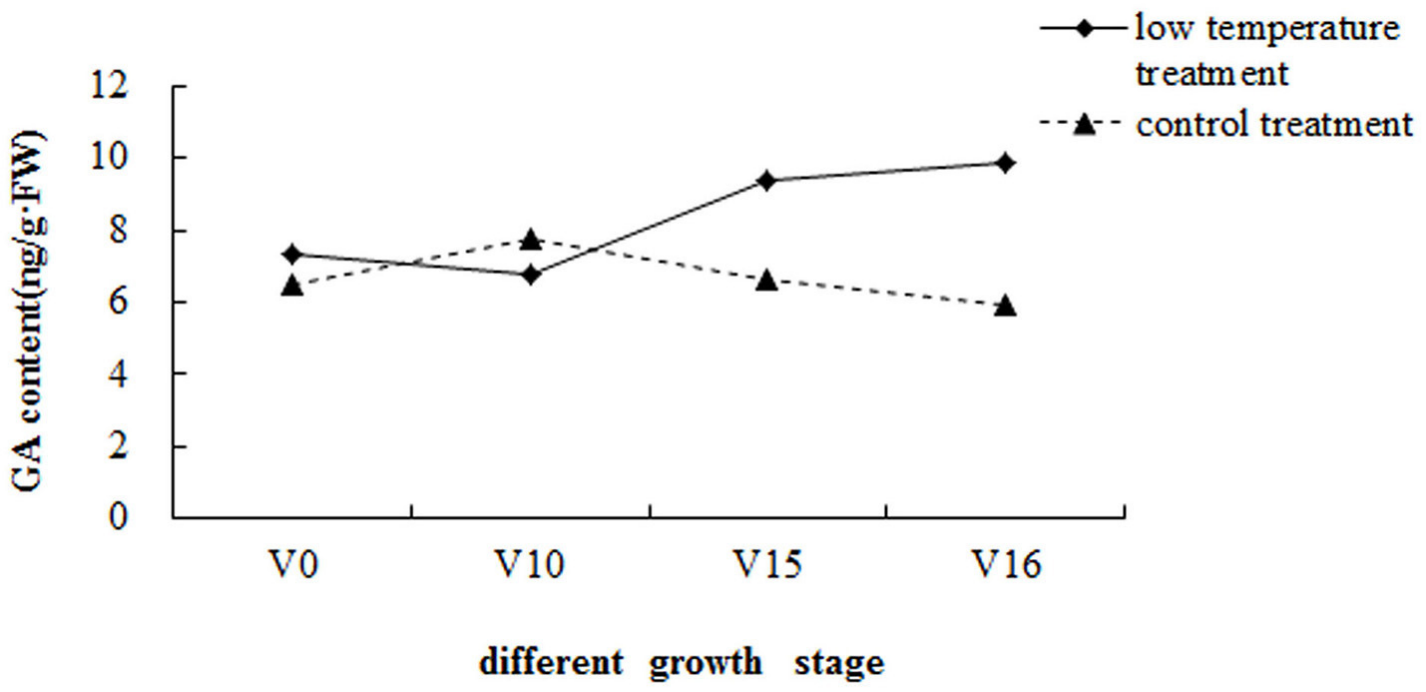
GA contents of pak choi shoot apices at different growth stages. V0, after low-temperature treatment and transplanting; V10, 10 days after transplanting; V15, 15 days after transplanting (floral bud differentiation is imminent); V16, 16 days after transplanting (floral bud differentiation stage 1).

The GA contents of the pak choi shoot apices at different growth stages were measured (Figure 1). After low-temperature treatment (V0), the GA content was 7.36 ng/g·FW, then decreased rapidly with vegetative growth and was approximately 6.80 ng/g·FW 10 d after transplanting (V10). Before floral bud differentiation (V15), the GA content increased rapidly to 9.34 ng/g·FW. Subsequently, the GA content increased slowly and reached 9.86 ng/g·FW at floral bud differentiation stage 1 (V16). Thus, the GA content first decreased and then increased after transplantation, peaking at floral bud differentiation stage 1. However, the GA content of the shoot apices in the control group did not show the same trend. In addition, the GA content in the low-temperature treatment group was significantly higher than in the control group, with the exception of 10 d after transplanting (V10 and CK10). This suggested that GA accumulation in the shoot apices can initiate floral bud differentiation, and low-temperature treatment can increase GA content.

### Quality assessment of the sequencing results

The expression profiles of genes in the pak choi shoot apices at different growth stages were analyzed by RNA sequencing. As shown in Table 1, a total of 11,111,314, 11,268,910, 10,849,774, 11,072,438, 10,709,754, and 10,169,936 reads were produced from the V0, CK0, V10, V15, V16, and CK16 samples, respectively; clean reads (those remaining after filtering out the adaptor sequences, contaminating sequences and low-quality reads) accounted for 87.09, 99.86, 99.79, 99.90, 99.86, and 99.86% of the total reads, respectively, indicating that the sequencing quality was excellent.

**Table 1:** Illumina DNA sequencing reads from six libraries and mapping results.

**Sample**	**Total reads**	**Clean reads**	**Mapped reads**	**Unique mapped reads**	**Multiple mapped reads**
V0	11,111,314	9,676,674 (87.09%)	8,025,626 (82.94%)	6,915,600 (86.17%)	1,110,026 (13.83%)
CK0	11,268,910	11,253,403 (99.86%)	9,543,109 (83.80%)	8,741,375 (91.60%)	801,734 (8.40%)
V10	10,849,774	10,827,162 (99.79%)	9,156,589 (84.57%)	8,556,760 (93.45%)	599,829 (6.55%)
V15	11,072,438	11,061,770 (99.90%)	9,411,660 (85.08%)	8,827,419 (93.79%)	584,241 (6.21%)
V16	10,709,754	10,695,085 (99.86%)	9,010,384 (84.25%)	8,454,786 (93.83%)	555,598 (6.17%)
CK16	10,169,936	10,156,073 (99.86%)	8,609,294 (84.77%)	8,009,239 (93.03%)	600,055 (6.97%)

To obtain gene expression information, high-quality clean reads from six libraries were aligned with the reference genome database. A total of 82.94, 84.80, 84.57, 85.08, 84.25, and 84.77% of the reads, including unique mapped reads and multiple mapped reads, were mapped to the reference genome for samples V0, CK0, V10, V15, V16, and CK16, respectively. The proportion of mapped genes was high, indicating that the sequences and reference genome are suitable for further analysis. The unique mapped reads accounted for 86.17, 91.60, 93.45, 93.79, 93.83, and 93.03% of total mapped reads in the six libraries for V0, CK0, V10, V15, V16, and CK16, respectively, and could therefore be used for further analysis.

### Expression analysis of genes encoding GA-metabolizing enzymes

Unlike the majority of GA, which is inactive, GA_1_ and GA_3_ have biological activity (Pan, 2008). GA is mainly synthesized in the stems and roots of plants, and a small amount is synthesized in mature leaves. It is not transported to other tissues. In cells, GA is synthesized in the plastids, endoplasmic reticulum and cytoplasmic matrix (Reinecke and Pharis, 2013). The KEGG pathway map of diterpenoid biosynthesis (ko00904) from RNA sequencing was shown in Supplementary Figure S1, in which the GA metabolism is marked in red. GA is synthesized in three steps from geranylgeranyl pyrophospha (GGPP) as a precursor. The first step occurs in the protoplast. GGPP forms copal pyrophosphate under the catalysis of CPS (5.5.1.13) and then forms ent-kaurene with catalysis by KS (4.2.3.19). The second step takes place in the endoplasmic reticulum with O_2_ as the substrate, NADPH as the cofactor (Rademacher, 2003), and KO (1.14.13.78) as the catalyst. Ent-kaurene undergoes constant oxidation, generating ent-Kaur-16-en-19-ol, ent-Kaur-16-en-19-al, and ent-Kaur-16-en-19-oate. Finally, GA12-aldehyde is produced under the catalysis of KAO (1.14.13.79). The third step is carried out in the cytoplasm. GA12-aldehyde forms different structures of GA under the catalysis of different enzymes. During this process, under the action of GA20ox (1.14.11.12), GA3ox (1.14.11.15) and cytochrome P450 monooxygenase (P450-1, 2, 3), GA12-aldehyde generates bioactive GA_1_ and GA_3_. Under the action of GA2ox (1.14.11.13), GA_1_ undergoes 2β -hydroxylation to form inactive GA_8_ and GA_34_. GA20ox and GA2ox play key roles in the entire process of GA metabolism.

Some genes encoding enzymes related to GA metabolism in pak choi were listed in Table 2. Seven enzymes involving 18 genes were found to participate in GA synthesis. The enzyme involved in the catabolism of GA is mainly GA2-oxidase, 11 genes were found encoding the enzyme in pak choi.

**Table 2:** Enzymes and their coding genes in GA metabolism (RPKM: reads per kb per million reads).

	**Enzyme ID**	**Enzyme**	**Coding gene in pak choi**	**RPKM**			**RPKM**			**RPKM**		
				**After low temperature treatment**			**After transplanting**			**floral bud differentiation**		
				**CK0**	**V0**	**Expression pattern**	**V10**	**V15**	**Expression pattern**	**CK16**	**V16**	**Expression pattern**
Biosynthetic	5.5.1.13	CPS	Bra000864	0.07	0.19	Up	0.23	0.28	Up	0.05	0.29	Up
			Bra036239	1.82	4.81	Up	2.68	1.26	Down	1.08	0.92	Down
	4.2.3.19	KS	Bra035120	5.11	6.55	Up	3.11	3.93	Up	5.10	5.55	Up
	1.14.13.78	KO	Bra009868	46.66	58.43	Up	13.92	15.26	Up	12.88	24.20	Up
	1.14.13.79	KAO	Bra005596	8.63	39.37	Up	11.25	6.73	Down	22.62	14.49	Down
			Bra015394	2.38	4.30	Up	3.59	6.71	Up	5.59	6.99	Up
	1.14.11.12	GA20-OX	Bra027106	0.00	0.00	–	0.00	0.00	–	0.00	0.00	–
			Bra019165	0.00	1.96	Up	0.41	1.37	Up	0.30	0.71	Up
			Bra013890	7.80	10.75	Up	0.76	1.38	Up	1.78	2.84	Up
			Bra028706	0.18	1.52	Up	0.25	0.00	Down	0.00	0.26	Up
			Bra009285	1.80	39.03	Up	0.87	3.74	Up	0.00	0.00	–
			Bra028277	1.79	1.52	Down	0.25	0.12	Down	0.00	0.13	Up
			Bra022565	0.00	3.57	Up	1.25	0.25	Down	0.95	4.64	Up
	1.14.11.15	GA3β -OX	Bra026757	0.00	0.00	–	0.00	0.13	Up	0.00	0.00	–
			Bra026122	15.54	3.25	Down	2.66	4.26	Up	6.36	12.18	Up
			Bra008481	0.00	0.00	–	0.00	0.00	–	0.00	0.00	–
			Bra008480	0.00	0.27	Up	2.67	2.81	Up	2.47	1.10	Down
			Bra020909	0.00	0.00	–	0.00	0.00	–	0.00	0.00	–
Catabolism	1.14.11.13	GA2-OX	Bra030500	106.71	40.28	Down	4.73	0.89	Down	0.48	1.21	Up
			Bra033324	42.55	86.31	Up	12.76	3.86	Down	1.93	0.76	Down
			Bra010802	29.90	27.05	Down	15.06	3.07	Down	3.17	5.56	Up
			Bra032354	8.79	10.39	Up	9.08	6.53	Down	4.93	12.24	Up
			Bra030187	5.52	5.48	Down	6.53	3.83	Down	3.39	3.50	Up
			Bra032238	0.00	0.00	–	0.45	0.30	Down	0.00	0.31	Up
			Bra035038	0.00	0.00	–	1.51	4.54	Up	1.48	2.18	Up
			Bra008362	0.44	0.00	Down	6.46	48.56	Up	7.01	2.53	Down
			Bra005415	0.00	0.00	–	0.00	0.00	–	0.00	0.00	–
			Bra020890	0.00	0.00	–	0.57	0.86	Up	0.16	0.15	Down
			Bra038780	0.21	0.58	Up	0.14	0.43	Up	0.47	0.29	Down

Based on the results of the gene expression profiles, the expression of genes encoding enzymes involved in GA metabolism were analyzed. The results showed that the expression of Bra000864 encoding CPS, Bra035120 encoding KS, Bra009868 encoding KO, Bra015394 encoding KAO, Bra019165 and Bra013890 encoding GA20ox were all up-regulated in CK0 vs. V0, V10 vs. V15, and CK16 vs. V16; that is, the gene expression in cold treatment sample were higher than the control after cold treatment and at floral bud differentiation stage 1. Furthermore, the gene expression in shoot apices was higher immediately prior to floral bud differentiation than in the vegetative growth stage. The expression of Bra009285 encoding GA20ox was up-regulated after low-temperature treatment and immediately prior to floral bud differentiation; however, its expression was not detected in shoot apices at floral bud differentiation stage 1, indicating that low temperature promoted its expression. Bra026757 encoding GA3ox was expressed only in immediately prior to floral bud differentiation; it was not expressed in CK0, V0, V16 or CK16. Thus, the expression of Bra026757 was lower in shoot apices at the vegetative growth stage than in the shoot apices immediately prior to floral bud differentiation.

The expression of some genes that participate in GA biosynthesis were irregular. For example, the expression of Bra036239 encoding CPS, Bra005596 encoding KAO and Bra008480 encoding GA3ox were up-regulated after low-temperature treatment, indicating that the low temperature promoted their expression. Bra028706 and Bra022565 encoding GA20ox were up-regulated after low-temperature treatment and during floral bud differentiation; that is, their expression were higher in the low-temperature treatment group compared to in the control after treatment and at floral bud differentiation stage 1. Bra028277 encoding GA20ox and Bra026122 encoding GA3ox were up-regulated in floral bud differentiation and down-regulated in CK0 vs. V0 and V10 vs. V15, indicating that their expression at floral bud differentiation stage 1 were greater than in the control. These results indicated that the high expression of Bra028706, Bra022565, Bra028277, and Bra026122 are beneficial for the flowering of pak choi.

The above results showed that the expression of genes involved in GA biosynthesis were up-regulated, indicating that GA synthesis was accelerated. The measured GA contents were higher in V0, V15, and V16 than in CK0, V10, and CK16, respectively, and the changes in gene expression were consistent with the changes in GA content.

Among the genes encoding GA2ox involved in GA catabolism, the changes in gene expression were irregular. For example, the expression of Bra030500, Bra010802, and Bra030187 were down-regulated after low-temperature treatment, indicating that low temperature inhibited their expression. The expression of Bra008362 was down-regulated after low-temperature treatment and in floral bud differentiation. The expression of Bra033324, Bra020890, and Bra038780 were down-regulated in floral bud differentiation but up-regulated in CK0 vs. V0 and V10 vs. V15. These results indicated that the expression of these genes were down-regulated, causing GA to decompose more slowly, thereby increasing GA content. This further explains the change in GA content observed in pak choi.

In addition, the genes Bra027106 encoding GA20ox, Bra008481, and Bra020909 encoding GA3ox involved in GA synthesis, and Bra005415 encoding GA2ox involved in GA decomposition were not expressed during vernalization, floral bud initiation and development.

### Identification of differentially expressed genes related to GA metabolism

According to the sequencing results, the expression of genes related to GA metabolism were analyzed in different periods (Table 2). After low-temperature treatment, 12 genes related to GA biosynthesis were up-regulated compared to the control, while two were down-regulated; three genes were up-regulated and four were down-regulated in the catabolic process. When the shoot apices immediately enter the floral bud differentiation, 10 genes related to GA biosynthesis were up-regulated compared to in the vegetative stage, whereas five were down-regulated; four were up-regulated, and six were down-regulated in the catabolic process. At floral bud differentiation stage 1, 10 genes related to GA biosynthesis were up-regulated compared to in the vegetative stage, while three genes were down-regulated; six gene were up-regulated and four were down-regulated in the catabolic process. In summary, after low-temperature treatment, more genes involved in GA synthesis were up-regulated than down-regulated during vegetative growth and at floral bud differentiation stage 1; for the genes taking part in GA catabolism, slightly more genes were down-regulated than up-regulated, except for at floral bud differentiation stage 1. This may have led to the higher GA contents in samples V0, V15, and V16 compared to in samples CK0, V10, and CK16. These results were coincide with the measured GA contents.

According to FDR < 0.01 and Fold Change ≥ 2, the differentially expressed genes (DEGs) were identified (Table 3). The DEGs related to GA metabolism were not found in floral bud differentiation stage 1 (V16) vs. the vegetative shoot apices (CK16). Four DEGs after low-temperature treatment (V0 vs. CK0) and three DEGs between V15 and V10 were identified by gene ontology (GO); all of them were involved in GA biosynthesis (GO:0009686) or catabolism (GO:0045487).

**Table 3:** Differentially expressed genes related to GA metabolism at different growth stages of pak choi.

**Stage**	**Coding gene in pak choi**	**Gene name**	**Homology gene ID in Arabidosis**	**log2FC**	**Gene annotation**
After low temperature treatment	Bra005596	*KAO2*	AT2G32440	2.115	GA biosynthetic process; KAO activity
	Bra009285	YAP169	AT5G07200	4.18	GA biosynthetic process; GA 20-oxidase activity
	Bra022565	GA20OX2	AT5G51810	3.58	GA biosynthetic process; flower development; GA acid mediated signaling pathway
	Bra030500	GA2OX6	AT1G02400	−1.44	GA catabolic process;
After transplanting	Bra033324	GA2OX6	AT1G02400	−1.69	GA catabolic process; C-19,C-20 GA-2βactivity
	Bra010802	ATGA2OX2	AT1G02400	−2.24	GA catabolic process;C-19 GA-βactivity GA-βactivity
	Bra008362	ATGA2OX1	AT1G78440	2.87	GA catabolic process;C-19 GA-βactivity

Bra005596, the *KAO2* homologous gene, was up-regulated after low-temperature treatment. In the second step of GA biosynthesis, in which kaurene generates ent-Kaur-16-en-19-ol, ent-Kaur-16-en-19-al, and ent-Kaur-16-en-19-oate followed by GA12-aldehyde under the catalysis of cytochrome P450 monooxygenase, KAO catalyzed ent-kaurenoic acid to form GA12-aldehyde, which belongs to the cytochrome P450 CYP88A subfamily (Helliwell et al., 2001a,b). The subcellular localization of *AtKAO1* and *AtKAO2* revealed that they located in the endoplasmic reticulum (Helliwell et al., 2001b), where the second stage of GA synthesis is carried out. In addition, *AtKAO2* was highly expressed in germinating seeds, flowers and fruit pods (Regnault et al., 2014). The expression of *KAO2* was up-regulated in pak choi, which was beneficial for early flowering.

Both Bra009285 and Bra022565, which encode GA20ox, were up-regulated after low-temperature treatment. Five homologous genes encode GA20ox in *Arabidopsis*: *AtGA20ox1, AtGA20ox2, AtGA20ox3, AtGA20ox4*, and *AtGA20ox5*. Bra009285 and Bra022565 corresponded to *AtGA20ox3* (YAP169) and *AtGA20ox2*, respectively. *AtGA20ox3* was expressed in dry seeds, imbibed seeds and silique (Yan et al., 2014) and was found to aid GA biosynthesis in multiple biological processes (Plackett et al., 2012). *GA20ox2* promotes floral bud formation and fruit pod growth. The over-expression of *GA20ox* can lead to early flowering (Sun, 2008). The up-regulation of Bra009285 and Bra022565 was speculated to contribute to early flowering in pak choi.

Among the genes encoding GA2ox involved in GA catabolism, the *GA2ox6* homologous genes Bra030500 and Bra033324 were down-regulated after low-temperature treatment and immediate differentiation, respectively. The *ATGA2ox2* and *ATGA2ox1* homologous genes were Bra010802 and Bra008362, respectively, which were down-regulated and up-regulated after transplantation, respectively. Active GA2-oxidase can be inactivated by 2β -hydroxylation, which is one of the main methods of regulating GA activity in cells (Rieu et al., 2008; Wiesen et al., 2016). In *Arabidopsis*, five genes encode GA2-oxidase: *GA2ox1, GA2ox2, GA2ox3, GA2ox4*, and *GA2ox6*. The over-expression of these genes in *Arabidopsis* causes dwarfing, reduces the GA content, and delays flowering (Wang et al., 2004). Similar results were also obtained in transgenic poplar (*Populus* L), rice and bean (Sakamoto et al., 2001; Busov et al., 2003; Appleford et al., 2007). In this study, the genes encoding GA2ox expressed were down-regulated after low-temperature treatment, which may have increased the content of active GA. The expression of Bra030500, Bra033324 and Bra010802 were down-regulated, which was consistent with the GA content. However, the expression of Bra008362 was up-regulated, which did not coincide with the GA measurements. Further analysis is required to explain this discrepancy.

### qRT-PCR verification

**Figure 2:**
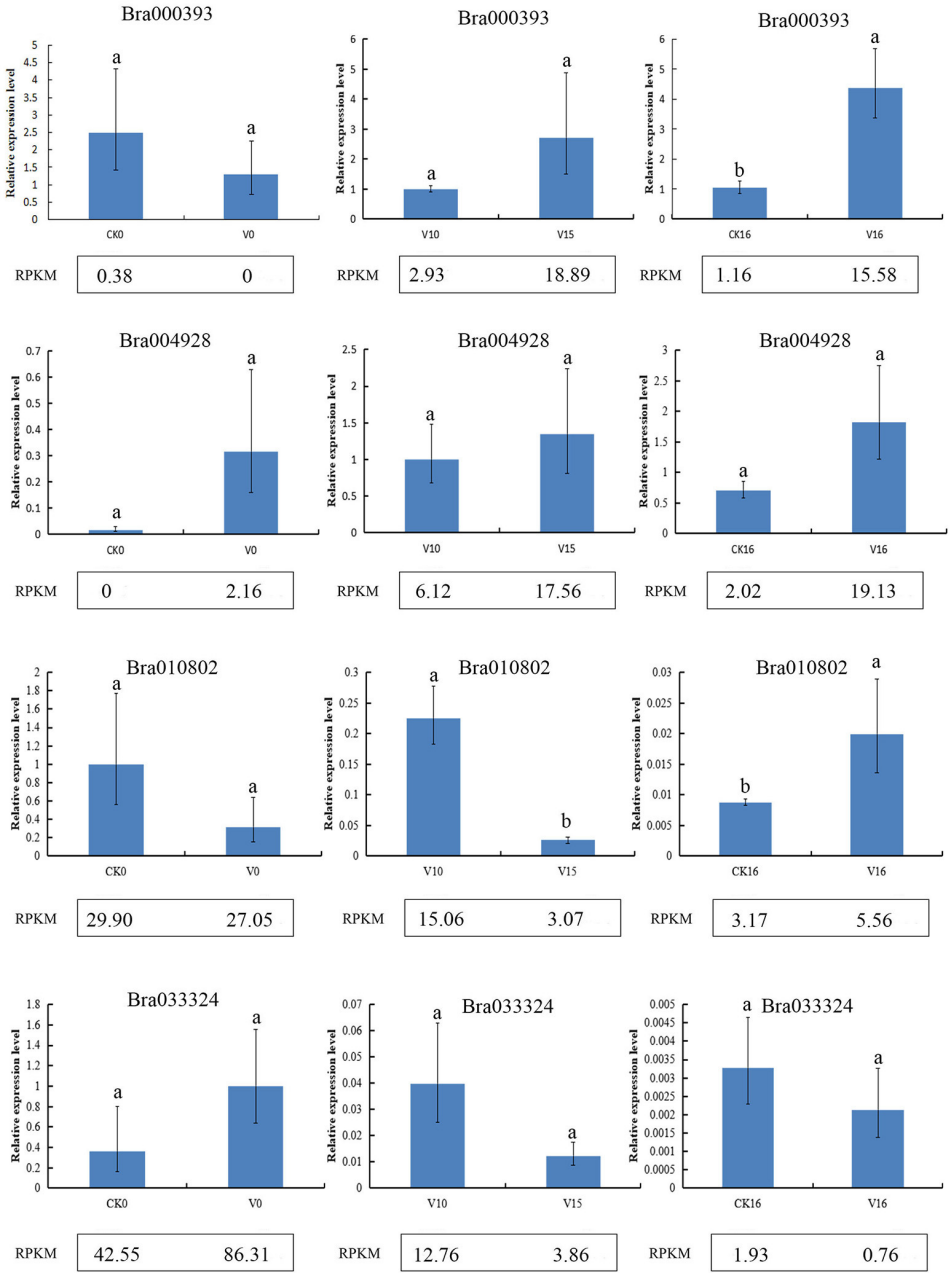
qRT-PCR analysis of RNA sequencing results. Different letters indicate a significant difference (*P* < 0.05).

To verify the reliability of the gene expression profiling, Bra000393, Bra004928, Bra033324, and Bra010802 were selected for qRT-qPCR verification in six samples (CK0, V0, V10, V15, CK16, and V16). The qRT- PCR results were consistent with the sequencing results, confirming the reliability of the sequencing results (Figure 2).

## Discussion

### Relationship between GA and vernalization, floral bud initiation and development

Hormones and GA in particular play an important role in plant vernalization, floral bud initiation and development. GA has been shown to induce the flowering of long-day plants under short-day conditions (Kinet, 1993; Buchanan et al., 2002). The GA content in germinating pak choi seeds treated at low temperature was approximately three times higher than that of the control (Li et al., 2002a). This suggested that vernalization treatment can promote the biosynthesis of endogenous GA, in agreement with the report of Burn et al. (1993). The results also suggest that vernalization can increase the activities of GA-biosynthetic enzymes and thus increase the content of GA. In broccoli, the GA content after low-temperature treatment was three times that in the control; the GA content then gradually decreased with green vernalization, reached a minimum at the critical period of floral bud differentiation, and then increased sharply (Jiang and Yu, 2004). The GA content was low in the vegetative growth stage of rape, increased rapidly during floral bud differentiation, and decreased sharply after floral bud differentiation (Li, 2010). For Chinese flowering cabbage, the GA content of the stem tip was highest in the vegetative period, decreased when floral bud differentiation was about to start, and then increased slightly when the floral bud began to differentiate (Li et al., 2002b). The variation of GA content in Chinese cabbage during floral bud differentiation was similar to that observed in Chinese flowering cabbage (Li, 2002). In summary, in brassicaceous vegetables, the GA content increases after cold vernalization, decreases when floral bud differentiation is imminent, and then gradually increases after floral bud differentiation. In this study, the GA content of pak choi was significantly higher after low-temperature treatment than in the control. The GA content decreased with vegetative growth and increased after floral bud differentiation, consistent with the results of previous studies. However, right before floral bud differentiation, the GA content was inconsistent. One possible explanation for this is that pak choi needs more GA at the beginning of floral bud differentiation.

### Expression of some important genes involved in GA metabolism

To further clarify the cause of the variation in GA content in pak choi during different growth stages, the GA metabolic pathway was analyzed. In the synthesis of GA, the conversion of GGPP to ent-kaurene is catalyzed CPS and KS. CPS is the key enzyme in GA biosynthesis. The oxidation of ent-kaurene to GA12-aldehyde is catalyzed by cytochrome P450 monooxygenase. The first three steps from ent-kaurene to ent-kaurene acid are catalyzed by KO (Helliwell et al., 1999). The following three steps from ent-kaurene acid to GA12-aldehyde are catalyzed by KAO (Helliwell et al., 2001b). Both KO and KAO belong to the cytochrome P450 monooxygenase family. The final step in GA biosynthesis is the formation of bioactive GA catalyzed by GA20-oxidase and GA3-oxidase. GA2-oxidase catalyzes the formation of inactive GA. These enzymes play important roles in GA metabolism and affect the level of active GA in plants, thereby affecting plant growth and development.

By analyzing the expression profiles, the changes in the expression of four genes encoding enzymes related to GA metabolism were found to be consistent with the changes in GA content. These genes were Bra035120, Bra009868, Bra015394 and Bra015394, and their *Arabidopsis* homolog genes encode *KS1, KO, KAO1*, and *GA20ox1*, respectively. In the first stage of GA synthesis, KS catalyzes the synthesis of ent-kaurene from copal pyrophosphate. In *Arabidopsis, AtKS1* was located in the chloroplast stroma by subcellular localization (Helliwell et al., 2001b). The loss of function of KS will lead to serious plant dwarfing and loss of reproductive capacity (Regnault et al., 2014). In this study, Bra035120 was up-regulated with the growth and development of pak choi, and its expression was consistent with the GA content of the shoot tips, in agreement with previous results. KO and KAO, which are members of the cytochrome P450 family of enzymes, participated in the second phase of GA biosynthesis. The process from ent-kaurene to ent-kaurene acid is catalyzed by *AtKO1*, which belongs to the cytochrome P450 CYP701A subfamily (Regnault et al., 2014). The up-regulation of *AtKO1* in transgenic *Arabidopsis* resulted in an increase in GA_4_ content (Xu et al., 2015). *KAO1* is highly expressed in the seeds and flowers of *Arabidopsis*, while its expression is low in seedlings and vegetative tissues (Xu et al., 2015). Bra009868 and Bra015394 have been suggested to be beneficial for the synthesis of active GA, which agrees with the results of this study. In *Arabidopsis*, the homologous gene of Bra013890 is *GA20ox1*, which belongs to the GA20ox family and is highly expressed in stems and flowers (Yan et al., 2014). *GA20ox1* plays an important role in the internode elongation of stamen filaments (Sun, 2008). The silencing of Sl*GA20ox1* does not contribute to pollen production in tomato (Olimpieri et al., 2011). Thus, Bra035120, Bra009868, Bra015394, and Bra013890 are thought to play important roles in the vernalization, floral bud initiation and development of pak choi.

Seven DEGs related to GA metabolism (Bra005596, Bra009285, Bra030500, Bra022565, Bra033324, Bra010802 and Bra008362) were identified by analyzing expression profiles. In *Arabidopsis*, their respective homologous genes are *KAO2, YAP169 (GA20ox3), GA2ox6, GA20ox2, GA2ox6, ATGA2OX2*, and *ATGA2OX1*, respectively. *KAO* mutations have been shown to cause severe stunting in a variety of plants including barley, rice, corn, peas and sunflowers (Helentjaris, 1995; Davidson et al., 2003; Sakamoto et al., 2004; Fambrini et al., 2011). *KAO* is thought to play an important role in the synthesis of GAs. *YAP169 (GA20ox3)* and *GA20ox2* are members of the GA20ox family. The over-expression of GA20ox leads to early flowering and stem elongation (Sun, 2008). *GA2ox6*, ATGA2OX2, and ATGA2OX1 encode GA2ox, and the over-expression of GA2ox can delay plant flowering and cause dwarfing (Sun, 2008). The over-expression of SlGA2ox1 leads to reduction in endogenous GA in tomato (Shen et al., 2016). GA3ox is also a key enzyme in GA biosynthesis, and its mutation leads to dwarfing and flower hypoplasia (Sun, 2008). DEGs encoding GA3-oxidase were not found in this study. It may be that GA20-oxidase and GA2-oxidase play important roles in regulating the level of active GA in plants, while GA3-oxidase has little effect on the late stage of GA biosynthesis in pak choi (Supplementary Figure S1). These results improve our understanding of the molecular flowering mechanism in pak choi.

## Conclusion

In this study, the GA content of the shoot tips at different stages in pak choi were measured. The results showed that low-temperature treatment increased the GA content, and enhanced GA accumulation initiated floral bud differentiation. To elucidate the molecular mechanism responsible for the changes in GA content, the expression of genes encoding enzymes involved in GA metabolism were analyzed based on the RNA sequencing. The results showed that the expression patterns of most genes involved in GA metabolism, particularly those of four genes (Bra035120, Bra009868, Bra015394, and Bra013890) coincided with the observed changes in GA content. In addition, by analyzing differentially expressed genes at different growth stages, seven genes (Bra005596, Bra009285, Bra022565, Bra008362, Bra033324, Bra010802, and Bra030500) were identified, and their expression were also consistent with the measured GA contents. These results contribute to the understanding of the molecular mechanism of changes in GA content during different developmental phases in pak choi.

## Author contributions

XW and MS performed the experiment, data analysis and prepared the manuscript. XQ and AP participated in performed the experiments. LH, GX, and GL participated in the experimental design. ML conceived the idea and participated in the interpretation of results and preparation of manuscript. JZ revised the manuscript. All authors read and approved the final manuscript.

### Conflict of interest statement

The authors declare that the research was conducted in the absence of any commercial or financial relationships that could be construed as a potential conflict of interest.

## Supplementary material

Supplementary Figure S1 Biosynthesis of diterpenoids (GA biosynthesis is marked in red).
