# Supplementary figures and images for: Genetic Regulation of GA Metabolism during Vernalization, Floral Bud Initiation and Development in Pak Choi (Brassica rapa ssp. chinensis Makino)
Source: Front Plant Sci. 2017 Sep 30;8:1533. doi: 10.3389/fpls.2017.01533 (PMC5628244; doi:10.3389/fpls.2017.01533)

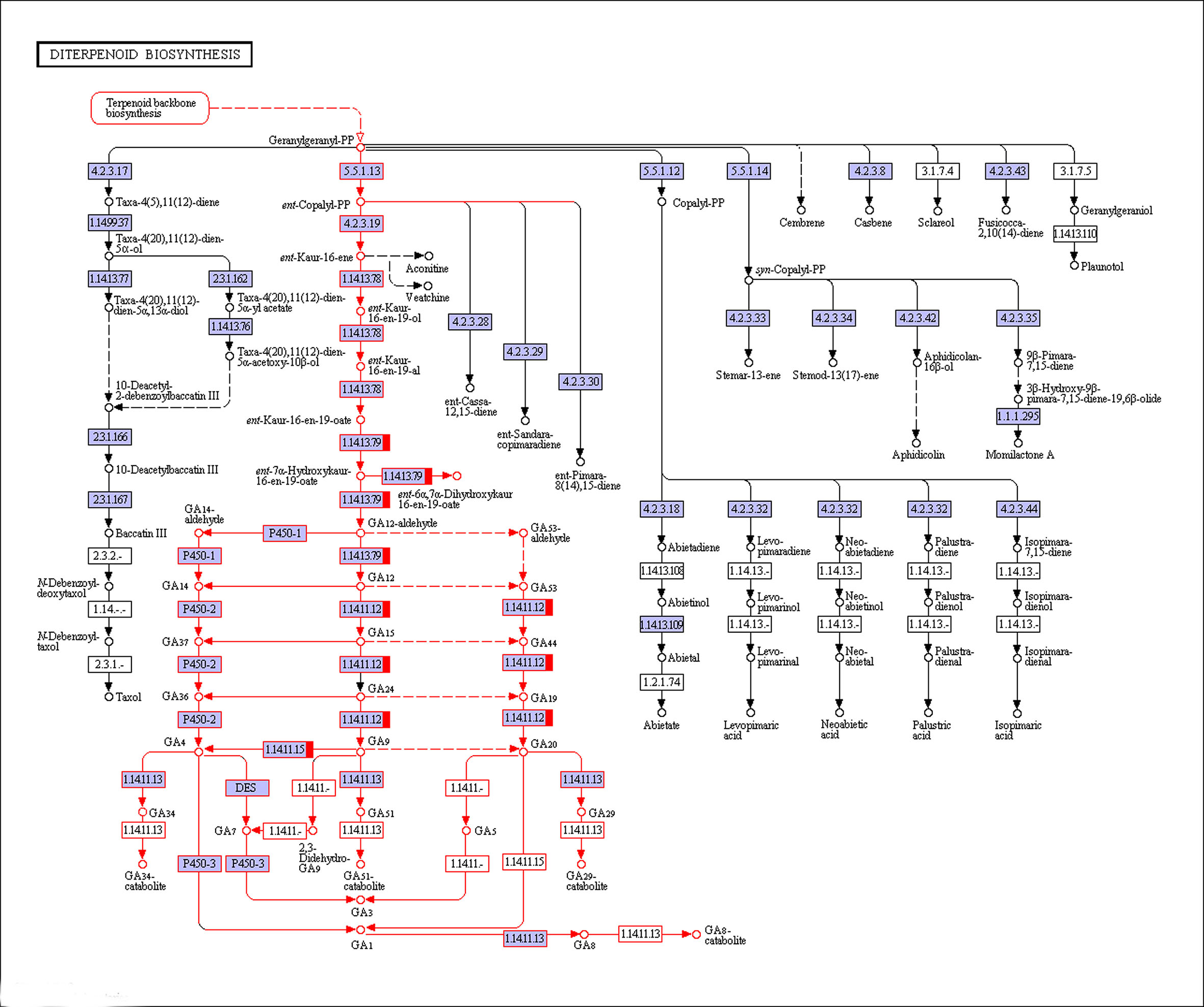

Supplement: Supplementary Figure S1 — Biosynthesis of diterpenoids (GA biosynthesis is marked in red). [file Image1.JPEG]
